# Factors influencing the stages of frailty among Korean older adults focusing on objective and subjective social isolation

**DOI:** 10.1186/s12877-022-03179-0

**Published:** 2022-06-07

**Authors:** Song Yi Han, Hye Young Jang, Young Ko

**Affiliations:** 1 Department of Nursing Science, Sunmoon University, 70, Sunmoon-ro 221 beon-gil, Tangjeong-myen, 31460 Asan-si, Chungcheongnam-do Republic of Korea; 2 School of Nursing, Hanyang University, 222 Wangsimni-ro, Seongdong-gu, 04763 Seoul, Republic of Korea; 3 College of Nursing, Gachon University, 191 Hambakmoeiro, Yeonsu-Gu, 21936 Incheon, Republic of Korea

**Keywords:** Older adults, Frailty, Social isolation

## Abstract

**Background:**

Although many studies have investigated the factors influencing frailty, few studies have confirmed the influence of social factors on the stages of frailty. This study was conducted to identify factors influencing the stages of frailty in Korean older adults, focusing on objective and subjective social isolation.

**Methods:**

This study analyzed the data of 10,041 older adults from the 2017 National Survey of Older Koreans. Two multiple logistic regression analyses were performed to identify the factors influencing the frailty stages. Frailty was calculated using the FRAIL scale with the five domains: fatigue, resistance, ambulation, illness, and loss of weight.

**Results:**

Among Korean older adults, 51.5% were in the robust stage, 42.5% in the pre-frail stage and 6.0% in the frail stage. As a multiple logistic regression analysis, participants with an intimate relationship (Odds ratio (OR) 0.93, 95% Confidential interval (CI) = 0.91–0.95) or objective social non-isolated participants were more likely to be in the robust group than the pre-frail group. Objective social-isolated participants were more likely to belong to the frail group than the pre-frail group: isolation from family member only (OR 1.57, 95% CI = 1.04–2.39), isolation from non-family member only (OR 1.75, 95% CI = 1.39–2.19), and isolation from both family and non-family member (OR 2.56, 95% CI = 1.67–3.92).

**Conclusions:**

This cross-sectional study showed that social isolation was associated with the stage of frailty. Therefore, researchers need to consider issues of social isolation of older adults in the development of frailty prevention and management intervention.

## Background

As the population ages, frailty has become an important public health issue [1]. In South Korea, rapid aging has increased the proportion of frail older adults with consequent social problems [2]. As a biological and physiological change that progresses with aging, frailty is defined as a decline in multiple body systems reserve [3, 4]. As frailty progresses in older adults, multiple functions such as physical, emotional and cognitive function decline [2, 5]. Therefore, the ability to maintain daily life independently gradually decreases [5]. This leads to dependence on others and increases the burden of the family and the society [6]. Frailty not only deteriorates the quality of life of older adults [7], but also leads to negative health outcomes such as institutionalization, hospitalization, disability, and death [1, 3, 8, 9]. Frailty is a continuum of robust, pre-frail, and frail stages, where vulnerability to poor health outcomes increases with progression [4, 10]. Therefore, the way to prevent the transition from the robust or pre-frail to frail stage is important [11].

According to previous studies, personal factors such as sociodemographic characteristics (e.g., low education level, advanced age [11–13], female gender [12, 14], and low household income [11, 12]), irregular exercise [4, 13], poor cognitive functions [14–16], low levels of physical function, and loss of sensory functions [14] have been identified as the risk factors of frailty. However, previous studies examining the relationship between social factors including social network size, social support and the progressing of frailty produced inconsistent findings [17–20].

Social isolation is multidemensional and can be divided into subjective social isolation and objective social isolation [21]. While the latter indicates the lack or the inadequacy of interactions with other people, such as limited size of social network or infrequent social contact, the former indicates the quality and perception of the relationships with others [21]. Older people are more likely to be isolated objectively or subjectively. Compared to younger generations, the social networks of older adults decrease in size [22] and are mostly composed of family and long-term friends [23]. Older adults may feel more loneliness and lack of intimate relationships by aging [24]. Such an increase of social isolation among older adults may be an important risk factor of frailty [17, 18]. However, only a few studies have investigated the correlation between the multidimensional properties of social isolation (subjective and objective social isolation) and the stages of frailty.

Therefore, this study aimed to explore the characteristics of subjective and objective social isolation and their correlation with the frailty stages in older adults residing in the community (Aim 1) and to identify the factors influencing the frail stages focusing on subjective and objective social isolation (Aim 2).

## Methods

### Study design

This study conducted a secondary analysis of the 2017 data from the National Survey of Older Koreans (NSOK) to investigate the factors influencing the stages of frailty in older adults living in community, focusing on objective and subjective social isolation.

### Data and ethical considerations

This study analyzed the 2017 data from the NSOK conducted by the Ministry of Health and Welfare [25]. The NSOK has been conducted every three years since 2008 to study older adults aged ≥ 65 years in South Korea. In 2017, 10,299 older adults aged ≥ 65 years in 934 regions were studied. The survey uses nationally representative samples of non-institutionalized Korean older adults aged 65 or over who lived in the community. The data can be obtained from the public data portal (data.go.kr) after the institution’s application and subsequent approval [25]. The data of 258 individuals with missing values among the 10,299 in the original data set were excluded, and 10,041 older adults were included in the analysis.

### Measurements

#### Frailty

Frailty was measured using the FRAIL Scale with the following five domains: fatigue, resistance, ambulation, illness, and loss of weight [26], which has been validated for use in older Koreans [27]. In this study, the five domains of the FRAIL Scale were assessed according to the following criteria. 1) Fatigue: For the question, “Have you experienced a significantly reduced level of activity or drive recently?”, a “No” response was given a score of 0, and a “Yes” response was given a score of 1. 2) Resistance: For the question, “How difficult is it for you to climb ten stairs without rest?”, a response of “Not difficult at all” or “A little difficult” was given a score of 0 (not difficult), and a response of “Very difficult” or “Too difficult to do” was given a score of 1 (difficult). 3) Ambulation: For the question, “How difficult is it for you to complete one round of a walk in a schoolyard (400 m)?”, a response of “Not difficult at all” or “A little difficult” was given a score of 0 (not difficult), and a response of “Very difficult” or “Too difficult to do” was given a score of 1 (difficult). 4) Illness: If a participant had three or fewer diseases diagnosed by a health care professional (e.g., hypertension, diabetes, cancer, chronic bronchitis or pulmonary emphysema, angina or cardiac infarction, other heart conditions, asthma, arthritis, stroke [paralysis or cerebral infarction], and chronic renal disease), a score of 0 was given. If a participant had been diagnosed with four or more diseases, a score of 1 was given. 5) Loss of weight: It was defined as loss of weight when an individual had a loss or gain of 5 kg without weight control over the previous six months and is underweight with body mass index (BMI) of less than 18.5 kg/m^2^. In this case, a score of 1 was given. A score of 0 was given to all other cases. The total score of the above five domains was calculated. A total score of ≥ 3 indicates frail, 1–2 indicates pre-frail, and 0 indicates a robust health state [28].

#### Social isolation

Objective social isolation was measured by combining the frequency of contact with family and contact with friends, neighbors, and acquaintances [17], based on two questions: “How often do you communicate (via phone, mobile message, email, letter, etc.) with a family member living elsewhere?” and “How often do you communicate with a friend, neighbor, or acquaintance?”. Responses were scored on a 7-point scale (1 = “never”, 2 = “1–2 times a year”, 3 = “1–2 times every 3 months”, 4 = “1–2 times a month”, 5 = “at least once a week”, 6 = “2–3 times a week”, 7 = “nearly every day (more than 4 times a week)”). We categorized the responses into the following 2 groups: not isolated (more than 4 times a week), 2–3 times a week, at least once a week, or 1–2 times a month) and isolated (1–2 times every 3 months, 1–2 times a year, or never). After regrouping, objective social isolation was categorized into the following four groups: 1) not isolated from both family and non-family member, 2) isolated from family member only, 3) isolated from non-family member only, and 4) isolated from both family and non-family member.

Subjective social isolation was measured based on the question: “With how many family members (parents and siblings), relatives, friends, neighbors, and acquaintances are you intimately close (to share all your thoughts and feelings)?”.

#### Sociodemographic and health-related characteristics

The sociodemographic factors included: gender, age, education level, annual household income, and living arrangement. Age was divided into a 65–74 years group and a ≥ 75 years group. Education level was reclassified into no formal education, elementary school graduation, and more than middle school graduation. Annual household income was divided into quartiles: lowest 25%, 26–50%, 51–75%, and highest 25%. Living arrangement was divided into living alone and living with others. Health-related characteristics including Activities of Daily Living (ADL) and Instrumental Activities of Daily Living (IADL) dependency, cognitive function, visual and hearing sensory functions, and exercise were assessed. ADL dependency was measured based on the seven items of the Korean Activities of Daily Living (K-ADL). IADL dependency was measured based on the ten items of the Korean Instrumental Activities of Daily Living (K-IDL). Dependency was assigned if at least one item indicated a need for assistance [29]. Cognitive function was measured using the Mini-Mental State Examination for Dementia Screening tool developed by Kim et al. [30]. This tool consists of 19 items, and the total score is calculated from the sum of all items. Normal cognition and cognitive decline are classified according to the norm score based on age, sex, and education level. The validity and reliability have been verified in previous studies, and the reliability of the tool was Cronbach’s alpha = 0.81 [30].

Visual and hearing sensory functions were assessed using questions of discomfort in daily life regardless of the use of assistants such as glasses and hearing aids. The questions were about discomfort while watching television, reading the newspaper, and talking on the phone or with someone next to them. The response of “Not uncomfortable” was categorized as “good” and “Uncomfortable or Very uncomfortable” as “not good”. We assessed exercise using two questions: "How many days per week do you exercise?" and "How many minutes do you exercise per day?". Individuals who exercised more than 30 min a day and more than 3 times a week were classified as “regular exercise”.

### Statistical analysis

SPSS 23.0 was used for all statistical analyses. To analyze characteristics of social isolation and the stages of frailty and the correlation between social isolation and the stages of frailty, χ^2^ test, t-test, one-way ANOVA with Scheffe test, and descriptive statistics were used (Aim 1). Two multiple logistic regression analyses were performed to identify the factors influencing the frailty stages, one with the “robust” group as the reference and the other with the “pre-frail” group as the reference (Aim 2). For these analyses, the model was run with five sociodemographic factors (gender, age, educational level, annual household income, and living arrangement), six health-related characteristics (ADL dependency, IADL dependency, cognitive decline, vision sensory, hearing sensory, and regular exercise), and two factors of social isolation (objective social isolation and subjective social isolation) in association with the stages of frailty. Odds ratios (ORs) indicated the likelihood of membership in the “pre-frail” group (relative to the “robust” group) and the “frail” group (relative to the “pre-frail” group).

## Results

### Differences in stages of frailty according to the characteristics of the participants

The general characteristics of the participants are presented in Table 1. The result showed that 51.5% of the participants were in the robust stage, 42.5% in the pre-frail stage, and 6.0% in the frail stage. Age, gender, education level, annual household income, and living arrangement were significantly associated with the stages of frailty (*p* < 0.001). ADL and IADL dependency, cognitive decline, visual and hearing sensory functions, and objective and subjective social isolation were significantly associated with the stage of frailty (*p* < 0.001) (Table 1).

**Table 1:** Stages of frailty according to sociodemographic and health-related characteristics

**Variables**	**Categories + (range)**	**Total**		**Stages of frailty**			**χ2 or F (p) (Scheffe test)**
				**Robust**	**Pre-frail**	**Frail**	
		**n (%) or M ± SD**		**n(%) or M ± SD**	**n(%) or M ± SD**	**n(%) or M ± SD**	
Total		10,041		5,171 (51.5)	4266 (42.5)	604 (6.0)	
Gender	Male	4277	(42.6)	2526 (48.8)	1590 (37.3)	161 (26.7)	194.926 (< .001)
	Female	5764	(57.4)	2645 (51.2)	2676 (62.7)	443 (73.3)	
Age (years)	65–74	5841	(58.2)	3502 (67.7)	2169 (50.8)	170 (28.1)	512.890 (< .001)
	≥ 75	4200	(41.8)	1669 (32.3)	2097 (49.2)	4359(71.9)	
Education level	No formal education	2376	(23.7)	838 (16.2)	1267 (29.7)	271 (44.9)	523.330 (< .001)
	Elementary school	3444	(34.3)	1700 (32.9)	1560 (36.6)	184 (30.4)	
	≥ Middle school	4221	(42.0)	2633 (50.9)	1439 (33.7)	149 (24.7)	
Household income (10,000won/year)	Q1 (≤ 686)	2489	(24.7)	1027 (19.9)	1255 (29.4)	207 (34.4)	304.215 (< .001)
	Q2 (687–991)	2528	(25.2)	1189 (23.0)	1136 (26.6)	203 (33.6)	
	Q3 (992–1,470)	2507	(25.0)	1361 (26.3)	1037 (24.3)	109 (18.0)	
	Q4 (≥ 1471)	2517	(25.1)	1594 (30.8)	838 (19.7)	85 (14.0)	
Living alone	No	7636	(76.1)	4145 (80.2)	3092 (72.5)	399 (66.1)	110.756 (< .001)
	Yes	2405	(23.9)	1026 (19.8)	1174 (27.5)	205 (33.9)	
ADL dependency	No	9356	(93.2)	5082 (98.3)	3946 (92.5)	328 (54.3)	1650.621 (< .001)
	Yes	685	(6.8)	89 (1.7)	320 (7.5)	276 (45.7)	
IADL dependency	No	7760	(77.3)	4621 (89.4)	3014 (70.6)	125 (20.7)	1638.457 (< .001)
	Yes	2281	(22.7)	550 (10.6)	1252 (29.4)	479 (79.3)	
Cognitive decline	No	8360	(83.3)	4455 (86.1)	3472 (81.4)	433 (71.7)	99.419 (< .001)
	Yes	1681	(16.7)	716 (13.9)	794 (18.6)	171 (28.3)	
Vision	Good	6651	(66.2)	3768 (72.9)	2596 (60.9)	287 (47.5)	251.178 (< .001)
	Not good	3390	(33.8)	1403 (27.1)	1670 (39.1)	317 (52.5)	
Hearing	Good	8249	(82.2)	4512 (87.3)	3360 (78.8)	377 (62.4)	285.023 (< .001)
	Not good	1792	(17.8)	659 (12.7)	906 (21.2)	227 (37.6)	
Exercise	Regular	5311	(52.9)	3212 (62.1)	2008 (47.1)	92 (15.2)	578.650 (< .001)
	Irregular	4730	(47.1)	1959 (37.9)	2258 (52.9)	512 (84.8)	
Objective social isolation	Not isolated from both family and non-family member	7559	(75.3)	4286 (82.9)	2993 (70.1)	280 (46.4)	590.259 (< .001)
	Isolated from family member only	597	(5.9)	283 (5.5)	277 (6.5)	37 (6.1)	
	Isolated from non-family member only	1626	(16.2)	529 (10.2)	857 (20.1)	240(39.7)	
	Isolated from both family and non-family member	259	(2.6)	73 (1.4)	139 (3.3)	47 (7.8)	
Subjective social isolation	Number of persons	2.28 ± 2.66		2.76 ± 2.91^c^	1.83 ± 2.27^b^	1.29 ± 2.07^a^	195.03 (< .001) (a < b < c)*

*M* mean, *SD* standard deviation, *ADL* Activities of Daily Living, *IADL* Instrumental Activities of Daily Living

*post hoc: Scheffe test (subgroup a= frail, b=prefrail, c=robust)

### Differences in social isolation according to the characteristics of the participants

Tables 2 and 3 show differences in social isolation according to the characteristics of the participants. We found differences in subjective social isolation by gender, age, education level, annual household income, and living arrangement, ADL dependency, IADL dependency, cognitive decline, and visual and hearing sensory functions (Table 2). Objective social isolation was significantly associated with gender, age, education level, annual household income, living arrangement, ADL dependency, IADL dependency, cognitive decline, and visual and hearing functions (Table 3).

**Table 2:** Subjective social isolation according to sociodemographic and health-related characteristics

Variables	Categories	M ± SD	t or F(p) (Scheffe test)
Gender	Male	2.34 ± 2.85	2.02 (.044)
	Female	2.23 ± 2.51	
Age (years)	65–74	2.63 ± 2.83	16.12 (< .001)
	≥ 75	1.80 ± 2.32	
Education level	None ^a^	1.46 ± 1.89	223.29 (< .001) (a < b < c)*
	Elementary school ^b^	2.15 ± 2.43	
	≥ Middle school ^c^	2.85 ± 3.05	
Household income (10,000won/year)	Q1 (≤ 686)^a^	1.77 ± 2.27	92.20 (< .001) (a < b < c < d)*
	Q2 (687–991)^b^	2.07 ± 2.45	
	Q3 (992–1,470)^c^	2.32 ± 2.68	
	Q4 (≥ 1471)^d^	2.95 ± 3.04	
Living alone	No	2.37 ± 2.73	6.39 (< .001)
	Yes	2.00 ± 2.40	
ADL dependency	No	2.34 ± 2.65	7.63 (< .001)
	Yes	1.51 ± 2.73	
IADL dependency	No	2.53 ± 2.75	20.13 (< .001)
	Yes	1.44 ± 2.11	
Cognitive decline	No	2.35 ± 2.65	5.71 (< .001)
	Yes	1.94 ± 2.67	
Vision	Good	2.51 ± 2.81	13.22 (< .001)
	Not good	1.82 ± 2.28	
Hearing	Good	2.42 ± 2.74	12.91 (< .001)
	Not good	1.65 ± 2.16	
Exercise	Regular	2.66 ± 2.87	15.62 (< .001)
	Irregular	1.85 ± 2.33	

*M* mean, *SD* standard deviation, *ADL* Activities of Daily Living, *IADL* Instrumental Activities of Daily Living

* post hoc: Scheffe test (subgroup a,b,c,d)

**Table 3:** Objective social isolation according to sociodemographic and health-related characteristics

Variables	Categories	Total%	Not isolated from both family and non-family member	Isolated from family member only	Isolated from non-family member only	Isolated from both family and non-family member	X2 or F(p)
Gender	Male	42.6	3189 (42.2)	276 (46.2)	685 (42.1)	127 (49.0)	8.28 (.041)
	Female	57.4	4370 (57.8)	321 (53.8)	941 (57.9)	132 (51.0)	
Age (years)	65–74	58.2	4614 (61.0)	436 (73.0)	651 (40.1)	140 (54.1)	300.56 (< .001)
	≥ 75	41.8	2945 (39.0)	161 (27.0)	975 (59.9)	119 (45.9)	
Education level	No formal education	23.7	1564 (20.7)	134 (22.4)	596 (36.6)	82 (31.7)	229.61 (< .001)
	Elementary school	34.3	2652 (35.1)	173 (29.0)	540 (33.2)	79 (30.5)	
	≥ Middle school	42.0	3343 (44.2)	290 (48.6)	490 (30.2)	98 (37.8)	
Household income (10,000won/year)	Q1 (≤ 686)	24.7	1676 (22.2)	189 (31.6)	524 (32.2)	100 (38.6)	199.21 (< .001)
	Q2 (687–991)	25.2	1867 (24.7)	143 (24.0)	427 (26.3)	91 (35.1)	
	Q3 (992–1,470)	25.0	1937 (25.6)	127 (21.3)	398 (24.5)	45 (17.4)	
	Q4 (≥ 1471)	25.1	2079 (27.5)	138 (23.1)	277 (17.0)	23 (8.9)	
Living alone	No	76.1	5914 (78.2)	357 (59.8)	1214(74.7)	151 (58.5)	152.31 (< .001)
	Yes	23.9	1645 (21.8)	240 (40.2)	412 (25.3)	108 (41.5)	
ADL dependency	No	93.2	7221 (95.5)	563 (94.3)	1354(83.3)	218 (84.2)	350.97 (< .001)
	Yes	6.8	338 (4.5)	34 (5.7)	272 (16.7)	41 (15.8)	
IADL dependency	No	77.3	6180 (81.8)	477 (79.9)	942 (57.9)	161 (62.3)	468.41 (< .001)
	Yes	22.7	1379 (18.2)	120 (20.1)	684 (42.1)	98 (37.7)	
Cognitive decline	No	83.3	6458 (85.4)	502 (84.1)	1212(74.6)	188 (72.6)	135.58 (< .001)
	Yes	16.7	1101 (14.6)	95 (15.9)	414 (25.4)	71 (27.4)	
Vision	Good	66.2	5172 (68.4)	423 (71.0)	909 (55.9)	147 (56.8)	110.72 (< .001)
	Not good	33.8	2387 (31.6)	174 (29.0)	717 (44.1)	112 (43.2)	
Hearing	Good	82.2	6364 (84.2)	516 (86.4)	1195 (73.5)	174 (67.3)	150.88 (< .001)
	Not good	17.8	1195 (15.8)	81 (13.6)	431 (26.5)	85 (32.7)	
Exercise	Regular	52.9	4258 (56.3)	323 (54.2)	637 (39.2)	93 (35.8)	189.64 (< .001)
	Irregular	47.1	3301 (43.7)	274 (45.8)	989 (60.8)	166 (64.2)	

*ADL* Activities of Daily Living, *IADL* Instrumental Activities of Daily Living

### Factors influencing the stage of frailty (prefrail vs. robust and frail vs. prefrail)

The multiple logistic regression analyses show that the difference in factors influencing the frail stages (Table 4). The subjective or objective social-isolated participants were more likely to belong to the pre-frail group than the robust group. Participants with an intimate relationship were more likely to be in the robust group than the pre-frail group (OR 0.93, 95% CI = 0.91–0.95). Simultaneously, the objective social -isolated participants were more likely to be in the pre-frail group: isolation from family member only (OR 1.42, 95% CI = 1.18–1.71), isolation from non-family member only (OR 1.49, 95% CI = 1.31–1.69), and isolation from both family and non-family member (OR 1.81, 95% CI = 1.33–2.47). Additionally, female gender, advanced age, low education level, low annual household income, and living alone were factors that significantly related to the pre-frail group. ADL and IADL dependency, cognitive decline, visual and hearing function decline, and irregular exercise were factors that significantly related to the pre-frail group (*p* < 0.05).

**Table 4:** Factors influencing the stages of frailty

**Variables**	**Categories**	**Pre-frail stage (reference: robust)**			**Frail stage (reference: pre-frail)**		
		**B**	**OR (95% CI)**	***p***	**B**	**OR (95% CI)**	***p***
Gender	Male						
	Female	0.33	1.39(1.26–1.53)	< .001	0.25	1.29(1.01–1.65)	.043
Age (year)	65–74						
	≥ 75	0.33	1.40(1.27–1.53)	< .001	0.11	1.11(0.89–1.40)	.360
Education level	No formal education	0.24	1.27(1.11–1.44)	< .001	-0.10	0.90(0.68–1.20)	.474
	Elementary school	0.23	1.26(1.14–1.40)	< .001	-0.20	0.98(0.75–1.29)	.884
	≥ Middle school						
Household income (10,000 won/year)	Q1 (≤ 686)	0.45	1.57(1.38–1.79)	< .001	0.21	1.23(0.90–1.69)	.186
	Q2 (687–991)	0.25	1.28(1.13–1.46)	< .001	0.29	1.33(0.97–1.82)	.075
	Q3 (992–1,470)	0.14	1.14(1.01–1.30)	.034	-0.10	0.90(0.64–1.26)	.550
	Q4 (≥ 1471)						
Living alone	No						
	Yes	0.12	1.12(1.01–1.25)	.038	0.14	1.15(0.92–1.43)	.222
ADL dependency	No						
	Yes	0.67	1.96(1.51–2.55)	< .001	1.28	3.59(2.86–4.51)	< .001
IADL dependency	No						
	Yes	0.63	1.88(1.66–2.14)	< .001	1.42	4.13(3.23–5.28)	< .001
Cognitive decline	No						
	Yes	0.26	1.29(1.14–1.46)	< .001	0.06	1.06(0.84–1.34)	.603
Vision	Good						
	Not good	0.25	1.29(1.17–1.41)	< .001	0.15	1.17(0.95 -1.43)	.136
Hearing	Good						
	Not good	0.20	1.22(1.08–1.38)	.002	0.28	1.33(1.06–1.65)	.012
Exercise	Regular						
	Irregular	0.37	1.45(1.33–1.58)	< .001	1.10	3.02(2.35–3.87)	< .001
Subjective social isolation	Number of persons	-0.07	0.93(0.91–0.95)	< .001	0.02	1.02(0.97–1.08)	.393
Objective social isolation	Not isolated from both family and non-family member						
	Isolated from family member only	0.35	1.42(1.18–1.71)	< .001	0.45	1.57(1.04–2.39)	.033
	Isolated from non-family member only	0.40	1.49(1.31–1.69)	< .001	0.56	1.75(1.39–2.19)	< .001
	Isolated from both family and non-family member	0.60	1.81(1.33–2.47)	< .001	0.94	2.56(1.67–3.92)	< .001
Hosmer–Lemeshow test		x^2^ = 13.310	df = 8	*p* = 0.102	× 2 = 9.852	df = 8	*p* = 0.276
Nagelkerke R^2^		.169			.324		

*OR* odds ratio, *CI* confidence interval, *ADL* Activities of Daily Living, *IADL* Instrumental Activities of Daily Living

Objective social-isolated participants were more likely to belong to the frail group than the pre-frail group: isolation from family member only (OR 1.57, 95% CI = 1.04–2.39), isolation from non-family member only (OR 1.75, 95% CI = 1.39–2.19), and isolation from both family and non-family member (OR 2.56, 95% CI = 1.67–3.92). Older adults who were female, had ADL dependency or had IADL dependency were more likely to be frail group than pre-frail group. Additionally, older adults who reduced hearing sensory and who did not regularly exercise were more likely to be frail group than pre-frail group (*p* < 0.05).

## Discussion

This study aimed to identify factors influencing the stages of frailty in Korean older adults, focusing on objective and subjective social isolation. We discuss these points and suggest their implications.

First, this study showed that 6% of Korean older adults were in the frail stage and 42.5% in the pre-frail stage. This was similar to the results of a study of Japanese older adults, which found 5.8% in the frail stage and 40.8% in the pre-frail stage [31], but indicated a significantly higher percentage of frail older adults compared to another study in Greece, where 1.5% of older adults were in the frail stage [12]. According to previous studies, the percentage of transition from pre-frail to frail is high [32]. It is crucial to provide interventions that prevent the transition between the stages of frailty, especially by identifying factors influencing the transition from the pre-frail to the frail stage.

Second, although a difference in subjective social isolation by the stage of frailty was observed in our study, subjective social isolation was associated with the pre-frail stage and not with the frail stage. This result is similar to the findings of a study that found no association between emotional support and the progression of frailty [19]. Previous research that subjective social isolation had a stronger correlation with emotional factors such as depressive symptoms and psychological distress than with physical health factors [22] supports the results of this study.

In this study, objective social isolation was a factor associated with the worsening of the frailty stages. This result was similar with those of previous studies that used objective social isolation as a social isolation variable [17, 18, 33]. The previous studies suggesting reduced social network outside of cohabitants and reduced social interchange were associated with decreased physical activity [34] and with occurred cardiovascular disease [35] also support our findings. Interestingly, a gradient in the influence of objective social isolation across the frailty levels was found in our study. This means that objective social isolation is more important in the older adults with pre-frail stage than in those with robust state. Therefore, health care providers should make efforts to maintain social networks and promote social interchange among older adults regardless of their frailty level to prevent the progression of frailty. However, careful caution is needed in interpreting the results, as the causal relationship between progression of the stage of frailty and social isolation cannot be confirmed in this study. Therefore, association between social isolation and the progression of frailty needs to be reconfirmed through a longitudinal study.

Third, the subjective and objective social isolation increased with higher age in this study. This was similar with a previous study which noted that the social network with family, friends, and neighbors is reduced as age increases [22]. This is not only the consequence of death of close person, retirement, and loss of social role. Older adults also tend to choose their social networks in order to strengthen positive relationships and minimize negative ones [22]. Those changes in social factors with increasing age can lead the worsening of frailty [17, 18, 33].

Korean older adults used to live in a traditional culture that valued familial relationships [36]. But in the age of nuclear family, younger generations place less importance on the family, to gradually diminish the family-centered culture [37]. It implies that family support is weakening and isolation from family is increasing. This study’s findings also showed that they are more likely to belong to the frail group than the pre-frail group when the participants were isolated from “both family and non-family member” or “non-family member only” than when they were isolated from “family member only”. Considering that the support system of the family is weakening, we need to focus on maintaining social relationships with non-family members.

Bigger social isolation was observed in the participants with lower socioeconomic position or with living alone. These results were similar with the findings in previous studies [38]. To improve frailty, priority targets for interventions that help maintain existing social networks or help them connect with new social networks should be older adults with low socioeconomic status or living alone.

Fourth, the subjective and objective social isolation was significantly associated with health-related characteristics including ADL dependency, IADL dependency, decline in sensory functions, cognitive decline, and irregular exercise. Those findings were in line with previous studies [39, 40]. In Korea, people aged 65 and over who are in vulnerable groups on the basis of socioeconomic position and health status are managed through the home visiting health service program of public health centers [41]. Approximately 9% of older people receive those services [42]. Several European countries and Japan have provided a preventive home visit service for older adults universally [43] and its effectiveness has been reported in several studies [44, 45]. Home visit nurses can regularly visit older adults living in the community to check their health status, monitor social networks, and provide interventions to maintain social networks by screening individuals at risk of social isolation [46]. Therefore, the home visit health service of public health centers needs to be provided universally.

Furthermore, reduced visual and hearing sensory functions were related to the pre-frail stage. The decline in hearing, in particular, was shown to be a risk factor for the frail stage. This result was similar to that of a previous study [14]. Reduced hearing ability is one of common symptoms among older adults [46] and poses a challenge to communication and social interactions with others, leading to social isolation [47, 48]. Likewise, in this study, older adults with reduced hearing and those with normal hearing showed a difference in subjective and objective social isolation. Routine monitoring of communication problems caused by hearing difficulty is important [48]. Health care providers need to evaluate whether hearing problems may cause further social isolation and need to educate acquaintances of older adults [48].

Older adults with irregular exercise were more likely to belong to the frail group than the pre-frail group. This was similar with those of previous studies suggested that regular exercise is a protective factor against frailty [13] and an effective intervention to prevent frailty [49]. In this study, subjective and objective social isolation showed a strong correlation with irregular exercise. Previous study has shown that the combination of reduced physical activity and increased social isolation further increases the probability of transition to the frail stage [50]. Social isolation may play a role in reduced physical activity of older adults. So, maintaining social networks can be a way to prevent frailty by means of making them to exercise.

This study showed that different factors have an influence on each stage of frailty. These results suggest that researchers should consider differences in factors that influence the stages of frailty among Korean older adults. However, this study also has the following limitations. First, it was a cross-sectional study, so we are not able to identify causal relationship between social isolation and frailty. Second, while a cognitive decline was shown to be an important factor in previous studies, it was not found to influence the stages of frailty in this study. This may be because the data analyzed in this study were obtained from community residents with an insufficient number of older adults with cognitive decline. Lastly, the subject of the 2017 NSOK are community-dwelling older adults. So, study findings are not generalized to older adults who are institutionalized or admitted.

## Conclusions

This study investigated the factors influencing the stage of frailty in Korean older adults, focusing on social isolation. The results showed that the pre-frail stage was associated with sociodemographic characteristics, health-related characteristics, and social isolation, while the frail stage was associated with female gender, ADL and IADL dependency, declining hearing, irregular exercise and objective social isolation. Based on the findings in this study, we suggest follows.

First, social isolation and frailty are related, and the association is stronger in frail older adults and in older adults isolated from both family and non-family members. Therefore, social isolation needs to be considered together in the prevention and management of frailty in older adults. Second, maintaining sensory function, especially hearing function which plays an important role in social relationships can be helpful. Health care providers need to evaluate whether hearing problems may cause further social isolation. Third, regular exercise was associated with the frail stage. Encouraging older adults to exercise regularly can be considered, even if they are in the pre-frail stage. Useful resources and support may make available to help the older adults maintain their social network and exercise regularly in the pre-frail stage. Lastly, a longitudinal cohort study should be conducted to identify the causality between the valuables and the factors influencing each step of the transition between stages of frailty. An intervention study should also be conducted on older adults in the pre-frail stage to verify the effects on delaying the transition to the frail stage.

## Data Availability

The datasets used and/or analyzed during the current study are available from the corresponding author on reasonable request.

## Abbreviations

ADL: Activities of Daily Living
BMI: Body mass index
CI: Confidence Interval
IADL: Instrumental Activities of Daily Living
IRB: Institutional Review Board
M: Mean
NSOK: National Survey of Older Koreans
OR: Odds Ratio
SD: Standard deviation
